# Communicatively Restricted Organizational Stress (CROS) on Campus: An Exploratory Investigation of Stress and Support among Predominantly White University Faculty

**DOI:** 10.3390/bs12090299

**Published:** 2022-08-23

**Authors:** Alice E. Veksler, Justin P. Boren

**Affiliations:** 1 Department of Communication, Christopher Newport University, Newport News, VA 23606, USA; 2 Department of Communication, Santa Clara University, Santa Clara, CA 95053, USA

**Keywords:** organizational behavior, stress, organizational communication, interpersonal communication, human health, effort–reward imbalance, well-being, burnout, social support, personal workplace relationships

## Abstract

Communicatively Restricted Organizational Stress (CROS) is a phenomenon characterized by real and/or perceived prohibitions against communicating with others about one’s organizational stressors. Given that CROS is marked by an inability to utilize social support, effects are often profoundly negative for the organizational members. However, the extent to which CROS functions similarly across similar types of organizations is unknown. In this exploratory project, the effects of CROS are investigated in a small sample (*n* = 41) of predominantly white university faculty. Conceptualizations of CROS argue that it is dependent both on the existence of stress and the presence of close and potentially supportive relationships. Provided that academia is a high-stress environment characterized by a strong likelihood of the formation of Personal Workplace Relationships (PWRs), CROS should be prevalent for this population and should lead to negative effects. Results indicated that CROS exists for university faculty and that its prevalence correlated negatively with measures of social support. Furthermore, CROS-associated distress is positively associated with perceived stress, burnout, and overcommitment and negatively associated with work well-being and job satisfaction. Although objective physiological measures of health were collected, the data were not able to be analyzed. The discussion focuses on implications and directions for future research.

## 1. Introduction

Organizational stress can impact health and well-being in myriad ways [1,2]. Decades of research in the fields of organizational communication and organizational psychology have documented this phenomenon, and researchers have argued a compelling case for investigating the correlates to and antecedents of negative work-stress-related outcomes [1,3,4,5,6]. One such variable of interest is Communicatively Restricted Organizational Stress (CROS), which is defined as “a perceived inability to communicate about a particular stressor” [7] (p. 34) that occurs in an organizational context and acts to exacerbate the distress associated with workplace stressors.

Past research indicates that the presence or absence of CROS can have meaningful impacts on individuals’ organizational lives, as well as on their overall health and wellbeing [7,8]. However, given that CROS is a somewhat newly identified phenomenon, it is not yet clear whether CROS functions similarly across different organizational settings. Furthermore, although the initial work on CROS hypothesized associations between CROS and both psychological and physiological markers of health, to date, only the former set of associations has been evaluated. Therefore, the purpose of this study is to present data from an exploratory data collection investigating how CROS functions in one specific environment expected to be particularly susceptible to CROS (i.e., a high-stress organization notable for fostering personal workplace relationships) and to evaluate associations between CROS and physiological markers of stress.

CROS is a phenomenon that causes individuals to feel a psychological or institutional pressure (which can be real or perceived) to not talk to others about their organizational stressors. The pressure can be explicit (for instance, in medical settings, HIPAA prevents providers from being able to speak openly about particular cases) or implicit. Implicit pressures can be due to the organizational culture or due to individual level factors, such as a reluctance to broach taboo topics. When organizational members experience stress, CROS exacerbates that stress in a number of ways. First, CROS prevents people from being able to directly address the issue because they feel that they cannot talk about it. Second, CROS prevents people from being able to access social support, affects coping, and frustrates their ability to convert perceived support into received support [7]. Research on CROS in various domains has shown associations between CROS and a number of correlates, including burnout, perceived stress, social support, organizational support, well-being, effort–reward imbalance, lost productivity, and general health [7,8,9].

### 1.1. CROS, Personal Workplace Relationships, and Academic Life

The effects of CROS rely on two factors. Firstly, CROS is dependent on the presence of personal relationships. The primary mechanisms of action for CROS are to impede the ability of workers to engage in socially supportive communication, to prevent perceived support from becoming enacted support, and to prevent instrumental support attainment in service of remediating the original stressor [7]. Yet, social support is a phenomenon that only exists within close social relationships [10]. Thus, for people to perceive a lack of support (and therefore CROS), the presence of close relationships is a necessary antecedent factor. To be clear, “personal relationships are an active, crucial ingredient in the social support equation” [10] (p. 41). Therefore, we expect that organizations with cultures that foster the development of voluntary interpersonal relationships among co-workers should be particularly sensitive to the effects of CROS as opposed to organizations marked more by loose ties or by role relationships where employees would be less likely to rely on one another for social support. Research on personal workplace relationships (PWRs) provides a framework for understanding this proposition [11]. The hallmarks of PWRs are that they are voluntary and consensual and are generally marked by stronger emotional connectivity than simple role-based relationships [11]. Additionally, PWRs allow for the very type of work–life bridging that creates the spaces within which socially supportive interactions live in organizational settings.

Unlike many other workplaces, the nature of faculty work makes academic settings especially fertile grounds for the formation of PWRs. As Moulin notes, “across a range of different laboratories and research groups and through their unique dynamics, it is usual to find friendships growing through the shared burden of long hours of work” [12] (p. 2). The organizational culture of universities strongly promotes formal and informal mentorship [13], and academics often enter into lifelong PWRs with mentors, mentees, and research collaborators in ways that are not often possible in other occupations [14]. Furthermore, PWRs are often formed as a means of establishing support to cope with workplace stressors [15]. The institution of tenure also fosters relationship development in part because faculty feel that they need relationships with colleagues in order to secure tenure and because once granted, faculty will often spend many years if not entire careers working with many of the same people [14]. Additionally, workplace romantic relationships are generally more accepted in academia than in other fields, and even though they are not promoted, cross-hierarchy romances are also commonplace [12]. The cohesive climates and bonds of colleagueship that are characteristic in academia [14] are precisely what makes university settings rife for PWRs.

Another potential locus for PWRs in higher educational settings is the faculty–student relationship [16]. Research suggests that it is not uncommon for faculty to engage in friendships, drinking relationships, and/or sexual and romantic relationships with students, which in turn have been shown to contribute to classroom incivility [16]. Provided that incivility is a source of stress [17] and that the taboo nature of some of these “multiple-role relationships” may give faculty pause about discussing them with others, this type of PWR may also be a source of CROS in academic settings. Furthermore, the specific nature of various PWRs themselves can also serve to be a source of both stress and CROS. For instance, workplace friendship deterioration and workplace romances are often stressors [11] that may also cause a perceived communication restriction, given the desire to shield coworkers from the vagaries of one’s personal life. Therefore, there are three ways that PWRs may lead to CROS: more opportunities for close relationships can lead to greater frustration when those individuals cannot be utilized for support; if the PWR itself is taboo, it can lead to self-imposed restriction on communicating about it; and if PWRs are themselves a source of stress, CROS can be a result.

Secondly, CROS is dependent on organizational stress. Therefore, one would expect to see a significant negative impact of CROS on workers in high-stress occupations. For example, recent research on CROS among working nurses suggests that nurses not only experience this phenomenon, but also report that CROS is associated with lost productivity, insomnia, and poor self-reported general health [9]. Thus, current evidence suggests that poor outcomes associated with CROS may be common in high-stress environments; however, further investigation is needed in other organizational contexts to support this proposition.

High workplace stress is well established for academics [18,19]. There are a number of sources of stress unique to academic settings that can lead to CROS. For instance, whereas evaluation is generally expected in the workplace, higher education is one of the few places where asynchronous anonymous feedback regularly flows hierarchically upwards from subordinate to superior, as is the case with course evaluations [20]. These student evaluations of teachers (SETs) can be extremely hurtful, affect identity and self-worth, and have material effects on factors such as merit pay and promotions [20]. Although relying on peer or supervisor support could help attenuate the stress associated with SETs [20,21], CROS, potentially due to feelings of embarrassment or shame [20], or institutionalized sexism and discrimination [21], may do the opposite and exacerbate the stressor. Additionally, the people most affected by discriminatory comments may also feel that they have fewer avenues to seek out support if they lack a sense of community at their institution.

Furthermore, job insecurity often associated with the uncertain expectations associated with being on the tenure track or with being in an adjunct/contingent position is also a source of stress in academia [22]. Since colleagues are often heavily involved with promotion and retention decisions, CROS may exist for those who do not want disclosure of their work difficulties or insecurities to affect such decisions.

Another persistent problem is classroom incivility [17], which can be a significant source of stress to the point of derailing careers and may also be subject to CROS. When topics become taboo, individuals may self-censor [7] and elect not to discuss the stressor with others. For instance, Boice argues that faculty often stay silent about classroom incivilities because of feelings of embarrassment or social impropriety, which frustrates their ability to address and resolve the issue [17].

Although emotional labor can be a significant component of many jobs, academic faculty are particularly affected due to the increasing mental health crisis on college campuses [23]. Faculty may feel implicit or explicit pressures to address their students’ mental health concerns due to the unique nature of the faculty–student relationship or because of institutions’ increased focus on retention. Although some faculty may be comfortable in this role, for others, it can be highly stressful due to a lack of preparation or qualifications to engage in such labor [23]. The increasing focus on diversity, equity, and inclusion efforts on campuses can also add to the emotional labor expected of faculty [24]. This is especially true for faculty from underrepresented groups and can be particularly stressful if that labor exposes faculty to racism or microaggressions [24]. Other stressors for faculty include work–life balance concerns, especially as they relate to uneven distributions of service responsibilities across demographic categories [22]; publishing demands; fundraising expectations [25]; teaching load [22,25]; and time pressures resulting in limited personal time [25].

In sum, the demands of academic life, including role conflict surrounding the tripartite teaching, research, and service model of tasks inherent in academics’ job descriptions, and the commodification of higher education (among other factors) lead to increased levels of burnout and organizational stress [19] as well as to depression, loss of productivity, and increased use of alcohol [18]. Many of these associations hold across cultures and across types of faculty appointments [26]. Although a number of individual and organizational level variables have been examined as correlates and antecedents of faculty burnout [19,26], to our knowledge, no research has evaluated the effects of CROS on burnout and other negative outcomes for faculty members. To examine these relationships, we aim to evaluate a battery of variables that are commonly noted as important for overall organizational and individual well-being.

### 1.2. Correlates of CROS

Stress is a physiological and psychological response to perceived strains or demands [1]. In the short term, stress can be adaptive in order to help an individual cope; however, over the long term, stress can accumulate and lead to dysregulation of various body systems, leading to allostatic load, all-cause mortality, and/or psychological damage [27]. Stressors can take many forms in organizational settings, including environmental/safety concerns, psychosocial pressures such as workplace conflict, and physical demands [7]. Given the numerous devastating effects stress can lead to [1], antecedents and consequents of stress are often evaluated in order to find targets for interventions that can reduce stress-related issues [28]. Given the conceptualization of CROS as a meta-stressor that functions to exacerbate the experience of the existing stress, we expect that workers’ perceived stress at a given point in time should be associated with CROS [7]. As workers experience more stress, CROS would be experienced as more distressing, which can in turn also lead to added perceptions of stress.

One of the most commonly assessed stress-related outcomes for employees is burnout [29]. Defined as the tripartite combination of emotional exhaustion, lack of professional efficacy, and cynicism (or depersonalization) [30,31], burnout is associated with a host of negative outcomes for both individuals and organizations [31]. Burnout-related effects include cardiovascular and immune system dysfunction [32], exhaustion, insomnia, and poor mental health at the individual level [33]. Organizationally, burnout has been associated with absenteeism, productivity loss, workplace injury, and workflow disruption [31]. Interventions aimed at reducing burnout aim to prevent and resolve the likelihood of these downstream effects. Provided that burnout is caused by a build-up of work stress [31,33] and that CROS frustrates one’s ability to address work stress [7], we expect that CROS should have strong positive associations with this variable. Furthermore, given that social relationships are of “critical importance” to burnout [33], a frustrating inability to harness the benefits of social relationships should be experienced as particularly upsetting.

Effort Report Imbalance and Overcommitment are two related and well-studied organizational variables [34]. The Effort–Reward Imbalance (ERI) model explains, through a social exchange framework, how stress is a function of organizational work [34]. ERI “asserts that the recurrent experience of failed reciprocity between high cost spent at work and low gain received in turn, activates sustained negative emotions of reward frustration and associated circuits of the brain reward system” [34] (p. 82). Accordingly, stress is produced when organizational members perceive that job efforts outweigh the potential for job rewards [34,35]. Additionally, individuals who have a regular imbalance due to excessive work might fall into a motivational pattern called overcommitment [34], which tends to lead to an amplification of the stress function due to their need for extrinsic approval of their overcommitment. Other research has found that CROS was connected to the ERI model among a sample of nurses, whereby highly stressed nurses who reported high CROS distress and low organizational support also had the highest reported amount of ERI [9]. This finding was evidence that CROS functions as a potential effort within the ERI model.

In an academic sample, we would surmise that faculty members often report high levels of efforts with low rewards, particularly depending on academic rank. For instance, long hours, heavy workload, work/life spillover, administrative work, poor compensation and benefits, and ambiguous career progression would all be considered efforts linked to a reduction in rewards [36]. For academics, most rewards come from informal mechanisms (e.g., peer and student interactions, subject passion, notoriety) and autonomy (i.e., either pedagogical and/or research autonomy) [36]. Given that many of these rewards are intangibles, they are not reported by all and might not overcome efforts [37]. Among academics, ERI and overcommitment have been associated with poor overall health [38], job strain, lowered job satisfaction, and intention to leave [37]. We believe that CROS would be associated with ERI and overcommitment, as individuals reporting more distress associated with CROS would report a greater imbalance and feelings of being overcommitted.

Sometimes referred to as the antipode of burnout [39], work well-being or engagement is a positive workplace variable. Although related to stress, satisfaction, burnout, coping, workplace relationships, and organizational conditions, well-being is a separate variable often conceptualized as the combination of vigor, dedication, and absorption [40]. Therefore, work well-bring encompasses resilience, energy, motivation, pride in one’s work, and engrossment [40]. If CROS functions as hypothesized, we should expect there to be a negative relationship between CROS and well-being, given that more open communication about organizational stress should be associated with more positive perceptions of the workplace.

Organizational members who are satisfied with their jobs generally report more positive outcomes [41]. For instance, job satisfaction is associated with lessened absenteeism [42], increased performance [43], lower stress [44], and better physical and mental health [41,45]. Job satisfaction or “…the cognitive evaluation of the well-being quality of one’s job such as with pay, co-workers or supervisors” [41] (p. 2) is therefore a variable of significant interest to organizational scholars. Although a multitude of factors, including intrinsic rewards, positive workplace relationships, and individual personality variables, have been identified to contribute to overall job-satisfaction [41], we posit that one’s ability (or inability) to communicatively address workplace stressors can be another such factor. Provided that job satisfaction is a meaningful subcomponent of overall life satisfaction [41] and is also associated with worker productivity [46], we feel a better understanding of what can affect perceived job satisfaction can help further elucidate this important variable.

According to organizational support theory [47], workers often engage in personification of their organization and deem it supportive/unsupportive, fair/unfair, or kind/unkind. These judgements are generally based on the actors within the organization, but attributed to the entity as a whole [47]. Perceived organizational support is associated with a host of benefits both for employees and organizations. Individuals experience need fulfillment, job satisfaction, and decreased stress [48]. At the organizational level, the entity benefits from greater worker commitment, lower absenteeism, and better performance [47,48]. Based on previous CROS research [8], we expect that faculty who perceive greater CROS should attribute that lack of support to the organization and therefore perceive lower levels of organizational support overall. Conversely, faculty who do not find their experience of CROS to be distressing are likely to perceive their institution as offering greater support to them.

The final variable of interest for the present study is social support or “the provision of care, resources, assistance, or information, before, during, or after times of burden or stress” [49] (p. 319). Decades of research on social support have led to the conclusion that it is a fundamental part of human existence and associates strongly with both physiological and psychological health outcomes [50]. Furthermore, as has been outlined above, social support is fundamental to the notion of CROS as a concept because CROS leads to a real or perceived impediment to the enactment of supportive interactions [7]. It is therefore our contention that those people who experience CROS should report a decrease in the amount of support they perceive to have not only at the organizational level (organizational support), but also across the three domains of friend/family, co-worker, and supervisor support.

Existing research on CROS, dating back to its initial conceptualization [7], suggests that CROS consists of two separate but related dimensions. First, CROS has to exist for a particular individual. That is, the person has to perceive that they are restricted in their ability to communicate about their organizational stress. The extent to which they feel restricted can range from feeling a complete inability to talk to others to something milder, where they feel discouraged or reticent but not fully unable to talk about these stressful things. This dimension is labeled CROS prevalence [8] and is a measure of the extent to which CROS is perceived to exist for a particular individual. Previous research suggests that for CROS to have an effect on an individual, they have to be at least somewhat aware of it [8]. Furthermore, we acknowledge that not all people in all organizations will perceive CROS and that different types of organizational settings may have more CROS prevalence than others. For instance, CROS prevalence for a national sample of working nurses averaged 3.60 (SD = 1.36) on a 1–7 scale, indicating moderate levels of CROS for that population [9], whereas CROS prevalence for a sample of graduate teaching assistants was quite a bit higher at 4.20 (SD = 1.43) [51].

However, the presence of CROS may not be sufficient to lead to negative outcomes. It is our contention that it is the extent to which a person feels troubled by the CROS that should relate to outcomes of interest. In other words, a person could feel restricted, even greatly so, but if that restriction does not bother them, we would not expect its mere presence to cause outcomes such as burnout or reduced job satisfaction. It may be, for instance, that people just accept CROS as a normative part of the organizational experience, and it therefore does not meaningfully impede upon their lives. This measure of the extent to which CROS is troubling is labeled CROS distress and is the second dimension of interest. Although it is important to establish the existence (prevalence) of CROS for a given population of interest, we propose that the distress dimension should be associated with the constructs introduced above, insofar as greater CROS distress should be associated with more negative experiences.

Interventions aimed at addressing faculty stress focus on ways to disrupt the causal chain of events that lead to negative outcomes [26], and therefore a better understanding of how stress functions for academics can better inform future intervention efforts. Provided the rationale above, we argue that academia is an important context for studying the effects of CROS because of a high-stress environment characterized by a strong likelihood of developing PWRs. Given the past research on CROS and associated outcomes, we extend the following research questions and hypotheses:

**RQ1:**  Do university faculty experience CROS?

**H1.**  *Among university faculty, CROS Distress is positively associated with perceived stress.*

**H2.**  *Among university faculty, CROS Distress is positively associated with burnout.*

**H3.**  *Among university faculty, CROS Distress is positively associated with overcommitment.*

**H4.**  *Among university faculty, CROS Distress is positively associated with Effort–Reward Imbalance.*

**H5.**  *Among university faculty, CROS Distress is negatively associated with well-being.*

**H6.**  *Among university faculty, CROS Distress is negatively associated with job satisfaction.*

**H7.**  *Among university faculty, CROS Distress is negatively associated with perceived organizational support.*

**H8.**  *Among university faculty, CROS Distress is negatively associated with social support.*

Although past research has evaluated the relationships between CROS and self-reported outcomes, there has been no evidence to date that CROS is associated with objective markers of health. Therefore, the second aim of this project was to extend the current body of knowledge on CROS to explore whether CROS is associated with known markers of physiological strain, as was originally proposed by Boren and Veksler [7].

Research on physiological markers of chronic stress in the social sciences generally focuses on the examination of a handful of variables that show consistent associations with stress and allostatic load [27]. Given that this is the first examination of CROS and physiology, the design of this project is purely exploratory, and therefore in lieu of formal hypotheses, we are guided by the following research question:

**RQ2:**  Is CROS Distress associated with any physiological stress markers among university faculty?

## 2. Materials and Methods

Data were collected at a mid-size liberal arts university on the West Coast of the U.S. and at a mid-size liberal arts university in the Southeast of the U.S. before the onset of the COVID-19 pandemic. Approvals for all study procedures were obtained from both institutions’ institutional review boards (IRBs) prior to the initiation of data collection.

### 2.1. Participants

Participants (*n* = 41) were university faculty members on and off the tenure track (Full professor, *n* = 9; Associate Professor, *n* = 6; Associate Professor—newly appointed, *n* = 2; Assistant Professor—applied for tenure and awaiting decision, *n* = 1; Assistant Professor = tenure track, *n* = 8; and non-tenure stream, *n* = 15). Participants were recruited by sending a study invitation through all-faculty campus email distribution systems. Participants were informed that the study would take approximate 30 min and that they would be compensated with $5 or a $5 gift card to the campus coffee shop. Interested faculty were directed to a screening questionnaire to determine eligibility. Participant distribution across locations was relatively equal (West Coast *n* = 19 and East Coast *n* = 22).

Due to constraints associated with the physiological assays, potential participants were excluded if they indicated that they had any current infection or illness, were taking certain medications, or reported discomfort with providing a saliva and/or blood sample through finger stick.

Of the 41 participants, *n* = 12 reported male sex assigned at birth, and *n* = 12 reported a male gender identity, whereas *n* = 29 reported female sex assigned at birth, and *n* = 29 reported a female gender identity. Ages ranged from 24 to 70 with a mean of 44.27 years (*SD* = 11.19). The majority of participants reported having a White/Caucasian/Euro-American ethnic background (*n* = 36), with others reporting Asian/Asian-American (*n* = 2), Black/African/African American (*n* = 2), Latino(a)/Hispanic/Mexican-American (*n* = 1), Native/Pacific Islander background (*n* = 1), and Non-Indian South Asian (*n* = 1) backgrounds. Total *n* = > 41 because participants were free to choose multiple options.

### 2.2. Procedures

Eligible and interested participants were invited to schedule a short appointment at one of the two locations’ laboratory spaces. Upon arrival, participants provided informed consent, and any questions were answered by one of the PIs. Participants were then instructed to follow standard protocols for providing blood and saliva samples for analysis of physiological stress effects, which took approximately 20 min.

Participants were then provided with a three-digit participant ID number to link their physiological data with their survey data and notified that a survey link (hosted online by qualtrics.com) would be emailed to them for completion later that day, and they were encouraged to complete it on the same day or soon after the lab visit. Participants completed a battery of self-report items as reported below, on their own time. There was no attrition, and all laboratory participants completed the survey portion of the study. Attention checks were included to ensure participants’ engagement with the survey.

### 2.3. Measures

All measures have been previously validated and showed adequate reliabilities.

#### 2.3.1. Communicatively Restricted Organizational Stress (CROS)

To evaluate CROS, we utilized Veksler and Boren’s [8] two-dimensional measure of CROS prevalence and distress (CROS-14), which is measured on a 7-point Likert-type scale ranging from “strongly agree” to “strongly disagree.” Sample items include “I feel that I am limited in my ability to talk about these stressful things” and “I feel anxious when I cannot talk about these stressful things.” Both dimensions were reliable in the present study (CROS Prevalence α = 0.85; CROS Distress α = 0.92).

#### 2.3.2. Perceived Stress

Stress was evaluated using the perceived stress scale (PSS-40) [52], which is measured on a five-point scale ranging from “never” to “very often”. A sample item asks, “In the last month, how often have you felt that you were unable to control the important things in your life?” The measure was reliable (PSS-4 α = 0.83).

#### 2.3.3. Burnout

The Maslach burnout inventory general scale (MBI-GS) [30] measures the three dimensions of burnout (emotional exhaustion, cynicism, and professional efficacy) on a 1–7 scale, where 1 = “very mild/barely noticeable” and 7 = “very strong/major”. Sample items include “I feel emotionally drained from my work” and “I doubt the significance of my work”. All of the dimensions had acceptable reliabilities (burnout emotional exhaustion dimension α = 0.92, burnout cynicism dimension α = 0.86, burnout professional efficacy dimension α = 0.78).

#### 2.3.4. Perceived Organizational Support (POS) and Job Satisfaction (OJS)

Organizational support and job satisfaction were measured using the items from the POS and job satisfaction index [53]. POS was measured using an eight-item unidimensional instrument assessed on a 5-point Likert-type scale (strongly disagree to strongly agree). Sample items include “Help is available from my organization if I have a problem” and “my organization cares about my well-being”. Job satisfaction (OJS) was measured on the same scale (sample item: “All in all, I am very satisfied with my current job”). Reliabilities for both were acceptable (POS α = 0.94; OJS α = 0.82).

#### 2.3.5. Work Well-Being

The Ultrecht work and well-being survey (UWES) [54] is a 17-item measure evaluating the vigor, dedication, and absorption dimensions of overall work-related fulfillment. Items were evaluated on a 7-point scale ranging from “never” to “always/every day.” Sample items include “I find the work that I do full of meaning and purpose” and “I am proud of the work that I do.” Based on recent guidance [55], a single composite score was calculated. The measure was reliable in this study (UWES α = 0.92).

#### 2.3.6. Social Support

Social support from supervisors, co-workers, and family/friends was measured using the multidimensional scales of perceived social support (SPSS) [56]. A sample item asks, “Each of these people can be relied on when things get tough at work.” The measure consists of the same four items for each relationship type and is measured on a Likert scale. Each dimension was reliable (SS-Supervisor α = 0.96; SS-Co-worker α = 0.88; SS-Family/Friends α = 0.92).

#### 2.3.7. Effort–Reward Imbalance (ERI) and Overcommitment

Effort–Reward Imbalance [57,58] is a measure of the relative effects of effort (e.g., “I have constant time pressure due a heavy workload”) compared to rewards (e.g., “I receive the respect I deserve from my superior or a respective relevant person”) as they relate to organizational work. The ERI short form uses 10 items (3 effort and 7 reward) measured on a four-point Likert-type scale (Strongly Disagree to Strongly Agree) to evaluate ERI.

The measure has been extensively validated [59]) and yields a ratio value wherein a score represents effort divided by reward multiplied by 7/3 [59]. Thus, a value of one on the ERI represents an equal distribution of efforts relative to rewards, whereas scores greater than one indicate more effort for each reward, and scores less than one indicate fewer efforts for each reward. Due to the ratio calculation used for this measure, an alpha is not computed for ERI.

Overcommitment was evaluated using the 6-item version of the measure [57], and includes items such as “I get easily overwhelmed by time pressures at work”, and was measured on a 4-point Likert-type scale ranging from “Strongly Agree” to “Strongly Disagree”. The overcommitment measure was reliable (α = 0.90).

## 3. Results

The first research question asked whether university faculty experience CROS. Results indicate that the participants in this study do experience CROS at moderate levels (*M* = 3.20, *SD* = 1.28). However, the results for CROS distress are quite a bit higher *(M* = 4.42, *SD* = 1.30), indicating that CROS distress is on the higher end of the spectrum for this sample, given that the score exceeds the hypothetical midpoint of the 1–7 scale. Research question one is therefore answered in the affirmative.

Provided the expected negative effects of CROS, the remaining hypotheses evaluated the associations between CROS and measures of interest to organizational scholars. Given the low sample size, parametric tests were not appropriate, so associations were evaluated using Spearman’s rho. All correlation coefficients and significance levels are reported in Table 1.

The first hypothesis predicted that CROS distress would be positively associated with perceived stress. This hypothesis was supported. The second hypothesis predicted that CROS distress would be positively associated with burnout for university faculty. As predicted, CROS distress was significantly and positively associated with the emotional exhaustion and cynicism dimensions of burnout and significantly and negatively associated with the professional efficacy dimension. Therefore, hypothesis two was supported. The third hypothesis predicated that CROS distress would be positively associated with overcommitment, which was supported by these data.

The fourth hypothesis predicated that CROS distress would be positively associated with Effort–Reward Imbalance. The data did not support hypothesis four. The fifth hypothesis predicated that CROS distress would be negatively associated with work well-being. The fifth hypothesis was supported. The sixth hypothesis predicted that CROS distress would be negatively associated with job satisfaction and was supported by these data. The seventh hypothesis predicted that CROS distress would be negatively associated with perceived organizational support. This hypothesis was not supported by these data. The final hypothesis (H8) predicated that CROS distress would be negatively associated with social support and was not supported.

The second research question asked whether CROS distress was associated with physiological stress markers. This research question remains unanswered. Analyses were conducted in consultation with University Statistical Consulting, LLC, using standard protocols. Data cleaning based on QNS (quantity not satisfied) readings, left and right censoring, and CV (coefficient of variation) thresholds yielded a sample size of *n* < 20, and additional data cleaning was no longer viable. As a result, inferential statistics would return biased results, and therefore no conclusions can be drawn from the physiological data.

## 4. Discussion

Communicatively restricted organizational stress (CROS) has been shown to be associated with a series of negative outcomes [8]. Identifying the factors that affect CROS can help target recommendations to those organizations that are most likely to be impacted so that negative outcomes could be potentially addressed by the organization. Past research has argued that CROS may be more prevalent in certain types of organizations, such as those recognized as “high stress” [9]. The present study identifies an additional factor that may make organizational members more susceptible to the negative effects of CROS. Specifically, we argue that the present findings suggest that organizations that are particularly likely to foster personal workplace relationships (PWRs) may also be more likely to lead to CROS and, importantly, make the experience of CROS especially problematic.

Results of this initial exploratory study indicate that the more distressing CROS is, the higher the likelihood of negative effects is. Specifically, the data indicate that CROS distress is associated with increased overall perceived stress (H1); greater burnout as evidenced by lower levels of professional efficacy and greater levels of cynicism and emotional exhaustion (H2); and greater overcommitment (H3). Furthermore, CROS distress was also associated with lower levels of work well-being (H5) and lower levels of job satisfaction (H6). Taken as a whole, these data demonstrate that CROS leads university faculty to feel worse about their workplace experiences. These findings have implications for overall health and well-being for faculty [1] but also for morale and retention [60].

These results also indicate that CROS does exist for university faculty, albeit at moderate levels, allowing us to draw conclusions about the first research question. More telling, however, is that the distress experienced as a result of CROS is high. In this sample, participants reported an average level of CROS distress that was greater than what is seen in a population level sample across a range of workplaces [8]. This suggests that something about the nature of this type of organization makes the experience of CROS especially distressing. We propose that high-stress work is one explanation but also that the nature of faculty work makes PWRs more likely [12], which, in turn, makes CROS distress and its effects more likely.

Although PWRs can lead to positive outcomes for faculty, such as more positive perceptions of work-family supportiveness [61], the mere presence of perceived support may not be adequate to temper the effects of job stress or restricted communication that faculty may feel. The results presented herein demonstrate that university faculty report relatively high levels of social support from supervisors and coworkers, with average levels all above the midpoint of the scale and greater than levels reported by a nationally representative sample of workers [8]. This indicates close relationships with colleagues and a greater likelihood of relying on co-workers and supervisors for support than in other workplace settings. This could indicate that faculty workplace relationships are closer and more intimate than other workplace relationships and could be a marker for the presence of PWRs [11].

We should note that three of our hypotheses did not receive support. There were no associations between CROS and effort–reward imbalance (H4), organizational support (H7), or social support (H8). It is unclear from the present data why there was no association between CROS distress and ERI or support, as was expected. One possibility is that the size of the sample prevented us from identifying relationships that may exist in actuality. The lack of findings may also be attributable to the fact that scale items did not focus on a specific stressor, which may have led to a disconnect between perceived CROS distress and perceived support.

A possible alternative explanation is that faculty may not want support from others or from their organizations when related to some types of workplace stress. Research on hurtful SETs indicates that some faculty do not seek out support and may instead utilize other methods of dealing with hurtful student comments [21]. They may also be reflecting on past experiences of negative support when thinking about experiences with co-workers. For instance, in the context of hurtful SETs, some faculty report that they feel socially undermined or experience negative affect when discussing their feelings [21]. Furthermore, even seemingly positive support can be unwanted if it threatens the recipients’ face needs, violates their privacy, or sets up an undesirable obligation [62]. Given the complex dynamics of faculty relationships, it is possible that faculty are wary of wanting to rely on certain others in the workplace for support if they fear negative associated outcomes [21,62]. In these cases, it would be reasonable to see no relationship between support availability and CROS distress because support is not seen as desirable for a given stressor, regardless of whether associated CROS distress is low or high. We are unable to test these speculations with the present data; however, this may be a promising direction for further research on the topic.

However, it should be noted that CROS prevalence was associated with decreased perceived organizational support, supervisor support, co-worker support, and ERI. It therefore appears more likely that, for university faculty, although CROS distress does not impact these variables, CROS prevalence does. This would suggest that regardless of how grievous faculty perceive CROS to be, its mere presence has an effect on perceptions of support and ERI.

This interpretation is understandable when viewed through the lens of PWR theorizing [11] because for those workplaces where interpersonal relationships are strong (and likely stronger than in other occupations), any amount of CROS, regardless of how distressing, should have a marked effect on people’s perceptions of their ability to rely on their colleagues and on their organization. In the absence of feelings of CROS, though, perceptions of support were quite high. In other words, when faculty feel comfortable speaking about their stress with colleagues, they are more likely to rely on them for support. The same pattern of findings is present for ERI, suggesting that CROS prevalence and CROS distress need to continue to be evaluated as separate but related factors.

We recognize that this was an initial exploratory study, and these associations should be reevaluated in larger samples. However, we believe that the present findings are notable and can help guide additional research. More generally, we believe these findings support the contention that workplaces that foster PWRs may be more susceptible to CROS than those where workers have looser ties to one another, and that the effects of this can lead to negative individual-level outcomes. This makes workplaces rife for PWRs an attractive target for intervention efforts aimed at reducing CROS [7].

Future research may be warranted to elucidate the specific stressors that cause CROS for such populations since this project did not address that crucial question. A better understanding of what causes CROS for specific sub-populations may help reduce the burden of this meta-stressor. For instance, organizational culture might have a large part to play in the way that CROS functions, especially among different groups. One particularly important avenue for future research on organizational culture relates to issues of implicit and explicit racism, sexism, and other forms of bias and discrimination that have documented effects on faculty experiences in academia [21] and can vary dramatically across institutions or even departments within the same university.

Additionally, future research can examine directionality, which we were unable to do with the present study design. For instance, it is not clear from these data whether CROS leads to reductions in perceived support, or whether a lack of support increases perceptions of CROS prevalence, or both. A better understanding of how these variables interact with one another can provide additional insight into how CROS functions for faculty and leads to downstream outcomes. Such investigation is especially warranted given the unexpected relationships we found between CROS prevalence and the support and ERI measures.

Finally, we should mention that the present project did not allow us to answer the research question, through which sought to evaluate whether CROS was associated with physiological health outcomes. We continue to believe that this is an interesting and important avenue for ongoing research on CROS. Research with larger samples is necessary in order to comport with best practices for cleaning data that would allow full confidence in the results while still adhering to the assumptions associated with parametric tests. Our analysis suggests samples of *n* = 300 or higher may be necessary.

In conclusion, the data presented in this initial exploratory study suggest that faculty relationships are likely marked by PWRs and that CROS leads to a reduction in perceived support availability within the academic workplace. When this CROS is distressing, it also then leads to other outcomes, such as burnout and reduced work well-being. Future research should retest these associations in a larger sample of faculty to confirm these initial findings. Furthermore, given that the present data came from mostly white faculty at predominantly white institutions, we would encourage future research to focus on representative faculty samples on more diverse campuses. Finally, we encourage additional research on the relationship between CROS and physiological outcomes.

## Figures and Tables

**Table 1:** Spearman’s rho coefficients, means, and standard deviations for all variables.

	1	2	3	4	5	6	7	8	9	10	11	12	13	*M*	*SD*
1. Prev	-													3.20	1.28
2. Dist	0.45 **	-												4.42	1.30
3. PSS-4	0.49 **	0.59 **	-											2.73	0.88
4. BO_EE	0.63 **	0.53 **	0.01 **	-										3.65	1.63
5. BO_CY	0.33 *	0.41 **	0.56	0.72 **	-									2.89	1.42
6. BO_PE	−0.17	−0.37 *	−0.51	−0.47 **	−0.51 **	-								5.55	0.91
7. POS	−0.52 **	−0.08	−0.02	−0.24	−0.14	0.09	-							3.55	0.85
8. OJS	−0.41 **	−0.42 **	−0.65 **	−0.62 **	−0.58 **	0.19	0.25	-						3.98	0.76
9. UWES	−0.12	−0.33 *	−0.43 **	−0.33 *	−0.50 **	0.55 **	0.03	0.37*	-					5.33	0.95
10. SS_Sup	−0.39 *	−0.06	0.12	0.01	0.05	−0.31	0.47 **	0.01	−0.19	-				3.71	1.24
11. SS_Co	−0.43 **	−0.03	−0.07	−0.22	−0.13	−0.19	0.67 **	0.27	0.10	0.47 **	-			3.87	0.82
12. SS_Fam	−0.23	0.08	−0.20	−0.19	−0.10	−0.01	0.21	0.08	−0.02	0.19	0.29	-		4.31	0.89
13. ERI	−0.48 **	0.19	0.41 **	0.58 **	0.40 **	−0.24	−0.56 **	−0.50 **	−0.05	−0.34 *	−0.44 **	−0.37 *	-	1.18	0.51
14. OC	0.57 **	0.40 **	0.51 **	0.75 **	0.49 **	−0.41 **	−0.23	−0.38 *	−0.18	0.00	−0.14	−0.16	0.55 **	2.71	0.73

Note. Prev = CROS Prevalence, Dist = CROS Distress, PSS-4 = Perceived Stress Scale, BO_EE = Burnout-Emotional Exhaustion Dimension, BO_CY = Burnout-Cynicism Dimension, BO_PE = Burnout-Professional Efficacy Dimension, POS = Perceived Organizational Support, OJS = Job Satisfaction, UWES = Work Well-being, SS_Sup = Social Support-Supervisor Dimension, SS_Co = Social Support-Co-Worker Dimension, SS_Fam = Social Support-Family Dimension, ERI = Effort–Reward Imbalance Ratio, OC = Overcommitment.* *p* < 0.05. ** *p* < 0.01

## Data Availability

The data that support the findings of this study are not publicly available due to ongoing analyses but are available from the corresponding author, A. Veksler, upon reasonable request.
